# A 52-week, open-label study evaluating the safety and efficacy of tabalumab, an anti-B-cell–activating factor monoclonal antibody, for rheumatoid arthritis

**DOI:** 10.1186/s13075-014-0415-2

**Published:** 2014-08-29

**Authors:** Maria Greenwald, Leszek Szczepanski, Alastair Kennedy, Melissa Veenhuizen, Wendy J Komocsar, Emery Polasek, Kelly Guerrettaz, Pierre-Yves Berclaz, Chin Lee

**Affiliations:** Desert Medical Advances, 72555 Fred Waring Drive, Palm Desert, CA 92260 USA; Wyzsza Szkola Spoleczno-Przyrodnicza, Lublin, 20-093 Poland; Private practice, 1300 36th Street #1A, Vero Beach, FL 32960 USA; Eli Lilly & Co, Indianapolis, IN 46285 USA; inVentiv Health Clinical, Princeton, NJ 08540 USA

## Abstract

**Introduction:**

The objective of this study was to evaluate the long-term safety and efficacy of tabalumab, a monoclonal antibody that neutralizes membrane-bound and soluble B-cell-activating factor, in rheumatoid arthritis (RA) patients.

**Methods:**

Patients with RA who completed one of two 24-week randomized controlled trials (RCTs) participated in this 52-week, flexible-dose, open-label extension study. Patients in RCT1 received intravenous placebo, 30-mg tabalumab or 80-mg tabalumab every 3 weeks, and patients in RCT2 received subcutaneous placebo or 1-, 3-, 10-, 30-, 60- or 120-mg tabalumab every 4 weeks (Q4W). Regardless of prior treatment, all patients in this study received subcutaneous 60-mg tabalumab Q4W for the first 3 months, then a one-time increase to 120-mg tabalumab Q4W (60-mg/120-mg group) and a one-time decrease to 60-mg tabalumab Q4W per patient was allowed (60-mg/120-mg/60-mg group).

**Results:**

There were 182 patients enrolled: 60 mg (*n* = 60), 60/120 mg (*n* = 121) and 60/120/60 mg (*n* = 1). Pretabalumab baseline disease activity was generally higher in the 60-mg/120-mg group. There was a higher frequency of serious adverse events and treatment-emergent adverse events, as well as infections and injection-site reactions, in the 60-mg/120-mg group. One death unrelated to the study drug occurred (60-mg/120-mg group). In both groups, total B-cell counts decreased by approximately 40% from the baseline level in the RCT originating study. Both groups demonstrated efficacy through 52 weeks of treatment relative to baseline pretabalumab disease activity based on American College of Rheumatology criteria improvement ≥20%, ≥50% and ≥70%; European League against Rheumatism Responder Index in 28 joints; Disease Activity Score in 28 joints–C-reactive protein; and Health Assessment Questionnaire–Disability Index.

**Conclusions:**

With long-term, open-label tabalumab treatment, no unexpected safety signals were observed, and B-cell reductions were consistent with previous findings. Despite differences in RCT originating studies, both groups demonstrated an efficacy response through the 52-week extension.

**Trial registration:**

ClinicalTrials.gov Identifier: NCT00837811 (registered 3 February 2009).

**Electronic supplementary material:**

The online version of this article (doi:10.1186/s13075-014-0415-2) contains supplementary material, which is available to authorized users.

## Introduction

B-cell-activating factor (BAFF) is a novel tumor necrosis family (TNF) family ligand that is necessary for B-cell survival [1]. Increased BAFF expression is also associated with autoantibody production and synovitis in rheumatoid arthritis (RA) patients [2]. Neutralizing BAFF may be an effective method of targeting B cells involved in RA pathogenesis.

Tabalumab is a human anti-BAFF monoclonal antibody that neutralizes both biologically active forms (membrane-bound and soluble) of BAFF [3] and has the potential to reduce RA signs and symptoms in patients in whom conventional treatments have failed [4-6].

This trial was designed to evaluate the long-term (52-week) safety and efficacy of subcutaneous 60-mg tabalumab administered every 4 weeks (Q4W), with an option to increase the dose to 120-mg tabalumab Q4W after 3 months of treatment, in patients with RA who had completed ≥24 weeks of participation and received at least three doses of study drug in a prior phase II tabalumab study.

## Methods

### Study design

In this 52-week, flexible-dose, open-label extension (OLE) study, we enrolled rheumatoid factor and/or anticyclic citrullinated peptide–seropositive patients with active RA (American Rheumatism Association 1987 revised criteria [7]) who had completed one of two phase II, 24-week, randomized controlled trials (RCTs) according to protocol. Patients from 14 countries were enrolled (Austria, Australia, Belgium, Brazil, Canada, Chile, Germany, Hungary, India, Mexico, Poland, Puerto Rico, Romania and the United States) [4,5]. Patients were excluded from participation if they had experienced a safety event during the prior RCTs that, in the investigators’ opinion, posed an unacceptable risk or if they had received any drug not allowed by the RCT protocol (ClinicalTrials.gov Identifier: NCT00837811). The study was approved by the local institutional review boards (see Additional file 1) in accordance with the Declaration of Helsinki and applicable laws and regulations. All patients provided voluntary written informed consent.

Patients enrolled in the first RCT (RCT1) were TNF antagonist inadequate responders (TNF-IR) who received placebo, 30-mg tabalumab or 80-mg tabalumab intravenously every 3 weeks for 6 weeks and were followed through week 24 [4]. During RCT1, patients with <20% improvement in tender or swollen joint counts (28 joints) by week 16 were given the option to receive an 80-mg tabalumab rescue dose at week 16. Patients enrolled in the second RCT (RCT2) were methotrexate inadequate responders (MTX-IR) who received placebo or 1-, 3-, 10-, 30-, 60- or 120-mg tabalumab subcutaneously Q4W for 24 weeks [5].

**Figure 1:**
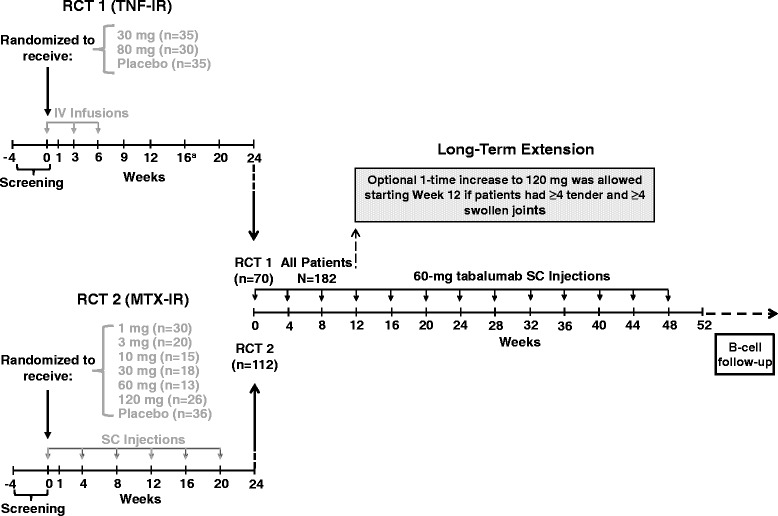
**Randomized controlled trial and open-label extension study designs.** ^a^Nonresponders (<20% improvement in tender or swollen joint counts in 28 joints) were eligible to receive an additional 30-minute infusion of 80-mg tabalumab as a rescue dose at week 16. IV, Intravenous; MTX-IR, Methotrexate inadequate responders; n, Number of patients per treatment arm; N, Total number of patients enrolled; RCT, Randomized controlled trial; SC, Subcutaneous; TNF-IR, Tumor necrosis factor inadequate responders.

All patients were on a stable background dose of methotrexate. During the OLE study, all patients received 60-mg subcutaneous tabalumab Q4W for 48 weeks, regardless of prior treatment (that is, tabalumab or placebo arm, dose of study drug or route of administration) (Figure 1). After 3 months of 60-mg tabalumab Q4W, each patient was allowed a one-time increase to 120-mg tabalumab Q4W based on the investigators’ clinical judgment and if the patient had at least four tender and at least four swollen joints. A one-time decrease to 60-mg tabalumab Q4W was allowed, but no further adjustments could be made.

### Assessments

Safety assessments included incidences of treatment-emergent adverse events (TEAEs), serious adverse events (SAEs) and assessments of vital signs, clinical laboratory data and immunogenicity. Blood samples were collected for determination of serum tabalumab concentrations and measures of biologic activity. Serum samples were taken after dose adjustments (increase to 120 mg or decrease to 60 mg). Measures of biologic activity included changes in C-reactive protein (CRP), total B cells (CD20 + CD3−), B-cell subsets (immature (CD19 + CD27 − IgD−), mature naïve (CD19 + CD27 − IgD+), switched memory (CD19 + CD27 + IgD−) and immunoglobulins (IgG, IgM and IgA). Infection rates and any association between infections and changes in B cells or immunoglobulins were also assessed.

Long-term efficacy was assessed at 24 and 52 weeks according to the proportion of patients with an American College of Rheumatology criteria improvement ≥20% (ACR20), ≥50% (ACR50) or ≥70% (ACR70), as well as on the basis of the ACR-N Index. Additional efficacy measures included the Health Assessment Questionnaire–Disability Index (HAQ-DI), the European League against Rheumatism Responder Index in 28 joints (EULAR28) and the Disease Activity Score in 28 joints (DAS28)–CRP.

### Statistical analyses

Safety analyses included all patients who received study drug. All efficacy and biologic activity analyses were conducted on an intent-to-treat basis and included patients who received at least one dose of study drug and had at least one postbaseline value. For ACR20, ACR50 and ACR70 analyses, patients who discontinued the study drug prior to week 52 were imputed as nonresponders (NRI; *n* = 34); otherwise, the last observed components of the index were utilized for each patient’s response (last observation carried forward (LOCF)). For HAQ-DI, EULAR28 and DAS28-CRP, the LOCF approach was used. Pharmacokinetic profiles were simulated, and a 90% prediction interval was created using the final model derived from RCT1 and RCT2 data. Graphical analysis was used for comparison of the model predictions with the observed OLE study data. Four patients in the 60-mg/120-mg group had no baseline B-cell data, and one patient in the 60-mg group had no postbaseline B-cell data. These patients were not included in B-cell analyses. One patient in the 60-mg/120-mg group was not included in absolute B-cell analyses because of an erroneous lab result that was not consistent with the patient’s absolute lymphocyte or absolute CD19 B-cell count. Two patients were excluded from efficacy analyses: one patient (60 mg) had no postbaseline efficacy data and was lost to follow-up, and one patient (60 mg/120 mg) was removed because of good clinical practice violations.

The expected maximum sample size was 249 patients. This sample size was not based on statistical considerations; it was expected that between 5% and 25% of patients from the originating studies would not elect to participate in the OLE study. Assuming 200 participants, the probability of observing at least one adverse event (AE) of a specific type is 0.866 when the background AE rate for the event is 1% in a similar cohort of patients during the time frame of this study.

Baseline data for demographics, clinical characteristics, biologic activity and efficacy measures were summarized from data collected before patients received the active drug in RCT1, RCT2 or the OLE study (that is, pretabalumab baseline). For patients assigned to receive tabalumab in RCT1 or RCT2, pretabalumab baseline data were collected at week 0 of the originating study. For patients assigned to receive placebo in RCT1 or RCT2, pretabalumab baseline data were collected either before receiving an optional 80-mg tabalumab rescue dose at week 16 in RCT1 or at OLE study entry. Data were descriptively summarized by treatment for all safety assessments at the endpoint and for biologic activity measures and efficacy measures at each visit.

## Results

### Patient disposition

Of the 186 eligible patients who completed RCT1 or RCT2, 98% (*N* = 182) were enrolled in the OLE study. Patients were evaluated within two different treatment groups: a 60-mg group (*n* = 60 comprising patients who received 60-mg tabalumab for whom the dose never escalated) and a 60-mg/120-mg group (*n* = 121, comprising patients whose dose was escalated from 60-mg to 120-mg tabalumab). The dose was escalated at week 12 for most patients (84 (69%) of 121) in the 60-mg/120-mg group. For one additional patient, the dose was escalated from 60 mg to 120 mg and then returned to 60 mg (60-mg/120-mg/60-mg group); this patient is not reported here.

Overall, 146 (80%) of 182 patients completed 52 weeks of treatment and 36 patients (20%) discontinued early. The most common reasons (≥5% of patients in either group) for early discontinuation were lack of efficacy (60 mg: 0%, 60 mg/120 mg: 9%), AEs (60 mg: 5%, 60 mg/120 mg: 5%) and patient’s decision (60 mg: 2%, 60 mg/120 mg: 6%).

### Baseline characteristics

Pretabalumab baseline RA activity levels were generally higher in the 60-mg/120-mg group (Table 1). Patients in whom the dose was escalated to 120 mg (60-mg/120-mg group) were found to have higher pretabalumab baseline values for the individual ACR criteria components (that is, tender and swollen joint counts, patient’s assessments of pain and disease activity and physician’s assessment of pain). The HAQ-DI, DAS28-CRP and CRP values were also observed to be slightly higher in the 60-mg/120-mg group than in the 60-mg group.

**Table 1:** **Pretabalumab baseline demographics and clinical characteristics** ^**a**^

	**Tabalumab (60 mg)**	**Tabalumab (60 mg/120 mg)**
	**(** ***N*** **= 60)**	**(** ***N*** **= 121)**
Males, *n* (%)	9 (15)	22 (18)
Age, yr	53 ± 12	52 ± 12
Duration of RA, yr	9 ± 7	**10 ± 8**
Swollen joint count (28)	10.2 ± 6.5	**12.2 ± 6.3**
Tender joint count (28)	13.1 ± 8.1	**16.1 ± 7.6**
Physician’s Global Assessment (VAS)	48 ± 21	**57 ± 22**
Patient’s Global Assessment of Disease Activity (VAS)	55 ± 23	**63 ± 23**
Patient’s Global Assessment of Pain (VAS)	55 ± 23	**60 ± 23**
HAQ-DI	1.5 ± 0.7	**1.7 ± 0.6**
CRP, mg/dl	2.2 ± 1.9	**2.3 ± 2.4**
DAS28	5.5 ± 1.3	**5.9 ± 1.1**
Prior TNF exposure, *n* (%)	17 (28)	**58 (48)**
Prior HCQ exposure, *n* (%)	25 (42)	**33 (27)**
Prior SSZ exposure, *n* (%)	14 (24)	**36 (30)**
Weekly dose of MTX, mg	16 ± 4	**16 ± 5**
Daily dose of prednisone dose, mg	7 ± 3	**7 ± 3**
Concomitant prednisone, *n* (%)	38 (64)	**92 (76)**

^a^CRP, C-reactive protein; DAS28, Disease Activity Score in 28 joints; HAQ-DI, Health Assessment Questionnaire–Disability Index; HCQ, Hydroxychloroquine; MTX, Methotrexate; RA, Rheumatoid arthritis; RCT, Randomized control trial; SSZ, Sulfasalazine; TNF, Tumor necrosis factor; VAS, Visual analogue scale. Data are mean ± SD unless noted otherwise. For patients assigned to receive tabalumab in RCT1 or RCT2, pretabalumab baseline data were collected at week 0 of RCT1 or RCT2. For patients assigned to receive placebo in RCT1 or RCT2, pretabalumab baseline data were collected either before patients received an optional 80-mg tabalumab rescue dose at week 16 in RCT1 or at open-label extension study entry. Numbers in bold denote pretabalumab RA activity levels that were greater for the 60-mg/120-mg tabalumab group compared to the 60-mg tabalumab group.

### Pharmacokinetics

Sample collection times and dosing regimens/history in the OLE study were considered to appropriately compare the data with pharmacokinetic profile model predictions. The mean number of tabalumab doses was comparable in the 60-mg and 60-mg/120-mg groups. During the OLE study, pharmacokinetic concentrations of tabalumab closely aligned with RCT1 and RCT2 model predictions (data not shown).

### Immunopharmacologic and disease-related biologic activity

Pretabalumab mean baseline CRP values were not significantly different in the 60-mg and 60-mg/120-mg group (2.2 ± 1.9 mg/dl and 2.3 ± 2.4 mg/dl, respectively) (Table 1). During the course of 52 weeks, mean (standard deviation (SD)) CRP values decreased in the 60-mg and 60-mg/120-mg groups (week 24: 1.4 ± 1.4 mg/dl and 2.2 ± 2.6 mg/dl; week 52: 1.3 ± 1.6 mg/dl and 1.6 ± 1.6 mg/dl, respectively). Median percentage changes in CRP from pretabalumab baseline in the 60-mg and 60-mg/120-mg groups, respectively, were −43% and −19% at week 24 and −47% and −29% at week 52.

**Figure 2:**
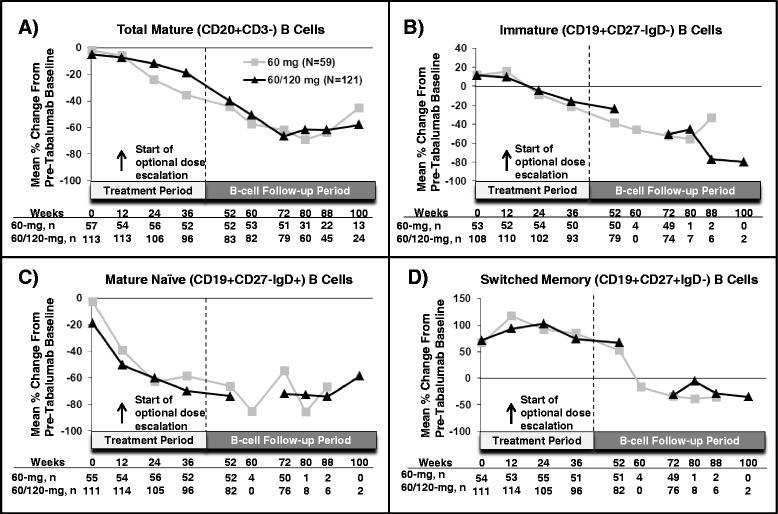
**Mean percentage changes in B cells and B-cell subsets.** Mean percentage changes in total mature (CD20 + CD3−) **(A)**, immature (CD19 + CD27 − IgD−) **(B)**, mature naïve (CD19 + CD27 − IgD+) **(C)** and switched memory (CD19 + CD27 + IgD−) **(D)** B cells are shown. The week 0 comparison is of the B cells to pretabalumab baseline counts before dosing with tabalumab. For patients who received tabalumab in the first or second randomized controlled trial, the pretabalumab baseline count represents B-cell count at a time point prior to week 0 of this open-label extension study. All patients were required to have B-cell follow-up through week 72; only patients who did not meet the definition of recovery were required to have follow-up beyond week 72. Data after week 100 are not shown, as there were fewer than five patients per arm. Ig, immunoglobulin; N, Number of patients per treatment arm; n, number of patients assessed at a given time point.

In all groups, total mature B-cell (CD20 + CD3−) and mature naïve B-cell (CD19 + CD27 − IgD+) counts gradually declined over time, but were not totally depleted at week 52 (Figures 2A and 2C). Immature (CD19 + CD27 − IgD−) and switched memory (CD19 + CD27 + IgD−) B-cell counts initially increased from pretabalumab baseline at week 12, followed by a gradual decline over time without total depletion at week 52 (Figures 2B and 2D). Switched memory (CD19 + CD27 + IgD−) B cells increased about 100% over baseline of the RCT originating study at week 12, then declined to approximately 60% to 70% over baseline values at week 52 (Figure 2D). Low total B-cell counts were defined as at least one B-cell assessment below 43 cells/μl and <50% of the pretabalumab baseline value. Of the 66 patients (36%) who completed 52 weeks of treatment and had low total B-cell counts, 49 completed follow-up and recovered by week 114, 66 weeks after the last injection. Recovery was defined as an absolute B-cell count ≥43 cells/μl or ≥50% of pretabalumab baseline. On the basis of the Kaplan-Meier estimate, the median time to recovery after last injection for these 66 patients was 40.6 weeks (confidence interval: 39.6 to 51.3).

**Figure 3:**
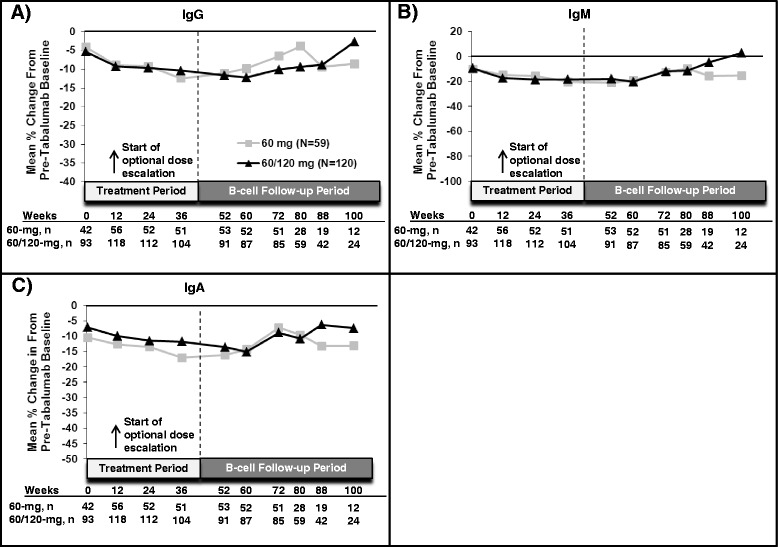
**Mean percentage changes in immunoglobulins.** Mean percentage changes in immunoglobulin G **(A)**, immunoglobulin M **(B)**, and immunoglobulin A **(C)** are shown. The week 0 comparison is of the immunoglobulins to baseline counts before dosing with tabalumab. For patients receiving tabalumab in the first or second randomized controlled trial, the baseline count represents the immunoglobulin level at a time point prior to week 0 of this open-label extension study. Data after week 100 are not shown, as there were fewer than five patients per arm. Ig, Immunoglobulin; N, Number of patients per treatment arm; n, Number of patients assessed at a given time point.

At the start of open-label treatment, both the 60-mg and 60-mg/120-mg groups showed a decline of 11% or less from their pretabalumab baseline serum immunoglobulin levels (IgG, IgM and IgA). At weeks 24 and 52, serum IgG, IgM and IgA continued to decrease from pretabalumab baseline for both groups (Figure 3). At week 36, the 60-mg group showed a more pronounced decrease in IgA levels than was observed in the 60-mg/120-mg group.

Treatment-emergent immunogenicity was defined as a fourfold increase from baseline titer or an increase of at least two dilutions during the treatment or follow-up period. Over the course of 52 weeks, 17 (9%) of 182 patients developed treatment-emergent antidrug antibodies (TEAbs); none were neutralizing. Persistent TEAb (positive antidrug antibody (ADA) detected at two or more sequential sample times with ≥12 weeks between the first and last samples or a positive ADA at the last obtained sample and no opportunity to assess persistence) was identified in 11 patients (6%). Two patients had persistent TEAb in RCT2 and remained positive for ≥12 weeks in the OLE study. Seven patients (4%) had follow-up emergent ADA (positive in the follow-up period only), and six were persistently positive. Plasma concentrations of patients who met TEAb criteria with tabalumab-specific ADA were compared to those of patients without ADA. Patients in the OLE study who had transient or persistent ADA had concentrations similar to those of patients without ADA and similar to the prediction interval (data not shown). The presence of ADA had minimal or no effect on the serum pharmacokinetics of tabalumab over the 52-week course of treatment.

### Safety

Three patients (5%) in the 60-mg group and six patients (5%) in the 60-mg/120-mg group discontinued during the OLE study because of an AE. In the 60-mg group, these events were myocardial ischemia, abdominal tenderness, and RA worsening (reported by one patient each). In the 60-mg/120-mg group, these events were intervertebral discitis, osteomyelitis, worsening of RA, breast cancer, cerebrovascular accident and pulmonary fibrosis (reported by one patient each).

A total of 133 AEs were reported for 182 patients (73%). The exposure-adjusted rates of TEAEs were 111/100 patient-years (PYs) for all patients while receiving 60-mg tabalumab and 118/100 PYs for all patients while receiving 120-mg tabalumab. The overall incidences of TEAEs were also higher in the 60-mg/120-mg group (79%) than in the 60-mg group (63%). Overall, the most frequently (≥5%) reported TEAEs were infection, RA worsening and injection-site reaction. The frequencies of these events in the 60-mg and 60-mg/120-mg groups, respectively, were infections (37% and 48%, respectively), RA worsening (18% and 25%, respectively) and injection-site reactions (3% and 12%, respectively). The exposure-adjusted rates of infection were 53/100 PYs for all patients while receiving 60-mg tabalumab and 62/100 PYs in all patients while receiving 120-mg tabalumab. Overall, the most frequently reported infection types were upper respiratory infections (URIs) and urinary tract infections (UTIs). URIs (including MedDRA (Medical Dictionary for Regulatory Activities) high-level terms: laryngitis, nasopharyngitis, pharyngitis, pharyngotonsillitis, rhinitis, sinusitis and URI) were reported with the same frequency in the 60-mg group (25%) and the 60-mg/120-mg group (25%). UTIs were reported in 3% of patients in the 60-mg group and 11% in the 60-mg/120-mg group. One patient (60-mg/120-mg group) reported a nonserious AE of fungal skin infection. Among patients with decreases in B cells (>50% reduction from pretabalumab baseline), the number of infections reported per person was similar to that of patients whose B cells did not fall below 50% of pretabalumab baseline (Table 2). Patients with reductions in serum IgG, IgM and IgA that fell below the lower limit of normal did not report a higher number of infections compared to patients with no reductions in the same serum immunoglobulins.

**Table 2:** **Percentage of patients with at least one infection during the treatment period** ^**a**^

	***n*** **/** ***N*** **(%)**	
	**60 mg (** ***N*** **= 60)**	**60 mg/120 mg (** ***N*** **= 121)**
B cells, cells/μl		
>50% reduction	10/38 (26%)	29/66 (44%)
≤50% reduction	9/16 (56%)	21/42 (50%)
No reduction	3/5 (60%)	6/10 (60%)
Serum IgG		
≥5.65 g/L	22/58 (38%)	56/118 (48%)
<5.65 to ≥4.24 g/L	0/1 (0%)	2/2 (100%)
<4.24 g/L	0/0 (0%)	0/1 (0%)
Serum IgM		
≥0.40 g/L	22/57 (39%)	55/113 (49%)
<0.40 to ≥0.30 g/L	0/2 (0%)	1/4 (25%)
<0.30 g/L	0/0 (0%)	2/4 (50%)
Serum IgA		
≥0.70 g/L	22/59 (37%)	58/120 (48%)
<0.70 to ≥0.525 g/L	0/0 (0%)	0/0 (0%)
<0.525 g/L	0/0 (0%)	0/1 (0%)

^a^Ig, Immunoglobulin; n, Number of patients with at least one infection with analyte measurement in that category; *N*, Number of patients with analyte measurement in that category; RCT, Randomized control trial. For patients assigned to receive tabalumab in the first or second randomized controlled trial (RCT1 or RCT2, respectively) pretabalumab baseline data were collected at week 0 of RCT1 or RCT2. For patients assigned to receive placebo in RCT1 or RCT2, pretabalumab baseline data were collected either before receiving an optional 80-mg tabalumab rescue dose at week 16 in RCT1 or at the time of open-label extension study entry.

SAEs also occurred more frequently in the 60-mg/120-mg group (13%) than in the 60-mg group (7%). In the 60-mg group, five SAEs occurred in four patients: myocardial ischemia and postprocedural hemorrhage (experienced by the same patient), RA worsening, hepatitis A infection and hip prosthesis dislocation. In the 60-mg/120-mg group, 21 SAEs occurred in 16 patients: hypertrophic cardiomyopathy; gastric ulcer; myocardial infarction; viral gastroenteritis and diabetic ketoacidosis (experienced by the same patient); intervertebral discitis and psoas abscess (experienced by the same patient); osteomyelitis; pneumonia; brain contusion, fall resulting in injury and RA worsening (experienced by the same patient); osteonecrosis and arteriosclerosis (experienced by the same patient); benign lung neoplasm and pulmonary fibrosis (experienced by the same patient); breast cancer; and cerebrovascular accident. Pulmonary fibrosis was reported in one additional 60-mg/120-mg patient, and RA worsening was reported in three additional 60-mg/120-mg patients. The one patient whose dose was escalated from 60 mg to 120 mg and then returned to 60 mg experienced mild pancytopenia and an ischemic stroke (reported on separate visits) during the 52-week treatment period.

One patient in the 60-mg/120-mg group died due to myocardial infarction (reported above as an SAE). The patient had received the first dose of study drug 182 days prior, was compliant with concomitant medications and had no history of coronary artery disease or cardiovascular disease other than hypertension and atherosclerosis. The investigator deemed the event not to be related to the study drug.

No notable differences or trends were identified in vital signs, electrocardiograms (ECGs) or chemistry, hematology and urinalysis panels. At the start of open-label treatment, the majority of patients in the 60-mg group (93%) and 60-mg/120-mg group (93%) had normal lymphocyte values; a small percentage had values that were low (60 mg = 3%, 60 mg/120 mg = 6%) or high (60 mg = 3%, 60 mg/120 mg = 1%). By week 52, lymphocyte values were in the normal range for 86% of the 60-mg and 88% of the 60-mg/120-mg patients. Relative to baseline, more patients had low lymphocyte values (60 mg = 11%, 60 mg/120 mg = 7%) at week 52.

### Clinical response

**Figure 4:**
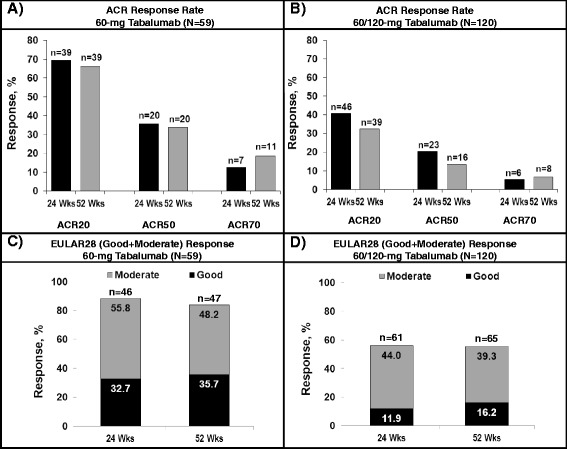
**Response rates on various efficacy parameters. (A)** and **(B)** ACR20, ACR50 and ACR70 response rates at week 24 (observed) and week 52 (NRI) for the 60-mg tabalumab group and the 60-mg/120-mg tabalumab group, respectively. **(C)** and **(D)** The EULAR28 (good + moderate) response rates for the 60-mg tabalumab and 60-mg/120-mg tabalumab groups, respectively, at week 24 (observed) and week 52 (LOCF). ACR, American College of Rheumatology Responder Index; EULAR28, European League against Rheumatism Responder Index based on 28 joints; LOCF, Last observation carried forward; n, Number of responders; N, Number of patients per treatment arm; NRI, Imputed as nonresponders; Wks, Weeks.

Both treatment groups demonstrated efficacy with 52 weeks of tabalumab treatment. The ACR20, ACR50 and ACR70 response rates in the 60-mg group at week 24 (observed; ACR20 = 70%, ACR50 = 36%, ACR70 = 13%) were generally maintained by week 52 (NRI/LOCF; ACR20 = 66%, ACR50 = 34%, ACR70 = 19%) (Figure 4A). Similarly, the ACR20, ACR50 and ACR70 response rates in the 60-mg/120-mg group at week 24 (ACR20 = 41%, ACR50 = 20%, ACR70 = 5%) were generally maintained by week 52 (NRI/LOCF; ACR20 = 33%, ACR50 = 13%, ACR70 = 7%) (Figure 4B). The mean (±SD) ACR-N in the 60-mg group was similar at week 24 (28.7 ± 53.6) and week 52 (31.9 ± 47.6). However, for the 60-mg/120-mg group, the mean (±SD) ACR-N was −8.9 ± 126.7 at week 24 (these patients were rescued during this period and dose-escalated). In this 60-mg/120-mg group, the ACR-N response improved to 11.3 ± 46.4 at week 52.

TNF-IR patients who originated from RCT1 had ACR20, ACR50 and ACR70 response rates of 40%, 17% and 6%, respectively, at week 24. Clinical improvement as measured by ACR response rates were observed for some patients at week 52 (based on NRI; ACR20 = 29%, ACR50 = 13%, ACR70 = 7%). MTX-IR patients who originated from RCT2 had ACR20, ACR50 and ACR70 response rates of 51%, 28% and 8%, respectively, at week 24. This response was maintained at week 52 (based on NRI; ACR20 = 52%, ACR50 = 24%, ACR70 = 13%).

At week 52, a good or moderate EULAR28 response was achieved by the majority of patients in the 60-mg group (47 of (84%) of 56) and the 60-mg/120-mg group (65 (56%) of 117) (Figures 4C and 4D). Reductions on the HAQ-DI (mean ± SD) from pretabalumab baseline were similar for the 60-mg group at week 24 (−0.25 ± 0.53) and week 52 (−0.27 ± 0.53) and for the 60-mg/120-mg group at week 24 (−0.26 ± 0.56) and week 52 (−0.30 ± 0.62). Reductions in DAS28-CRP scores (mean ± SD) from pretabalumab baseline were consistent over the course of 52 weeks of treatment in the 60-mg group (week 24: −2.0 ± 1.5, week 52: −2.1 ± 1.5) and the 60/120-mg group (week 24: −1.2 ± 1.4, week 52: −1.3 ± 1.5).

## Discussion

In this 52-week OLE study, we evaluated the long-term safety and efficacy of subcutaneous tabalumab in RA patients who had completed one of two RCTs.

Long-term tabalumab treatment resulted in gradual declines in total B cells without total depletion. Declines in mature naïve B-cell and immature B-cell subsets were also observed, whereas switched memory B cells initially increased from baseline of the RCT originating study and then declined by week 52. The majority of patients with low B cells at week 52 completed follow-up, and all patients who completed follow-up recovered. Median time to recovery after last injection was 40 weeks. There were no differences between dose groups in observed immunoglobulin reductions, except at week 36, when the 60-mg group showed a more pronounced decrease in IgA levels than the 60-mg/120-mg group. There was no indication that reductions in B cells or in serum immunoglobulins below the lower limit of normal were associated with an increased frequency of infections. Overall, these B-cell findings are consistent with tabalumab’s mechanism of action and the results of prior tabalumab studies. Other B-cell-targeted therapies, such as belimumab and atacicept, have been shown to reduce B cells incompletely [8,9], whereas, in contrast to the findings in the present study, rituximab has been shown to reduce CD19+ B cells to 0, often for months to years [10].

Tabalumab serum concentrations aligned closely with RCT1- and RCT2-derived model predictions, suggesting time-independent pharmacokinetics during the OLE study. Additionally, tabalumab treatment was associated with a low incidence of ADA, and none of the antibodies were positive in the neutralizing antibody assay, nor were they associated with changes in pharmacokinetics or the occurrence of AEs.

No unexpected safety signals were observed in this study, and slightly higher frequencies of TEAEs and SAEs were observed in the 60/120-mg than the 60-mg group. The higher frequency in the 60/120-mg group may be attributed to a dose–response effect. Overall, the frequency of TEAEs (range, 63% to 79%) and the frequency of SAEs (range, 7% to 13%) were consistent with other OLE studies of RA treatments such as belimumab, tocilizumab and abatacept [9,11,12]. In the present OLE study, exposure-adjusted rates of TEAEs were lower than rates reported for other biologics used to treat RA, although data in the previous studies were summarized from a greater number of PYs [13,14].

Over the course of 52 weeks of treatment, the results obtained for RA patients in both groups demonstrated efficacy on various measures of disease activity, including ACR20, ACR50, ACR70, EULAR28, DAS28-CRP and HAQ-DI. In the 60-mg/120-mg group, differences on the ACR-N from week 24 to week 52 that may be attributed to dose adjustment were noted, as the dose for the majority of these patients was increased from 60-mg to 120-mg tabalumab at week 12. In general, the dose given to patients with higher disease activity levels at pretabalumab baseline was escalated from 60 to 120 mg relative to patients whose dose was never escalated.

Interpretation of the efficacy and safety of tabalumab based on this study is limited by the absence of a control group, by small sample sizes (especially during B-cell follow-up) and by the heterogeneous population (TNF-IR and MTX-IR) who were on background methotrexate as well as other background medications in some instances. Additionally, the extent of tabalumab exposure varied, as patients were enrolled from two different RCTs involving a range of doses, dosing regimens and administration routes.

## Conclusion

Despite prior treatment differences, efficacy responses were observed in both groups with up to 52 weeks of open-label tabalumab treatment. Although a higher frequency of SAEs and severe TEAEs were observed in the 60-mg/120-mg group, no unexpected safety signals were observed and the frequencies of these events were within ranges reported for other biologics used to treat RA [9,11-14]. The reductions in total B cells were consistent with those observed in prior tabalumab studies. After this study was completed, phase III clinical trials were undertaken using tabalumab in RA patients. These trials were discontinued after interim analyses produced results that did not meet efficacy expectations [15,16]. No safety concerns were noted.

## Additional file

Additional file 1
**Ethical review boards that provided approval for this study.**

## Abbreviations

ACR: American College of Rheumatology
ACR20: American College of Rheumatology criteria improvement ≥20%
ACR50: American College of Rheumatology criteria improvement ≥50%
ACR70: American College of Rheumatology criteria improvement ≥70%
ADA: Antidrug antibody
AE: Adverse event
BAFF: B-cell-activating factor
CRP: C-reactive protein
DAS28: Disease Activity Score in 28 joints
ECG: Electrocardiogram
EULAR28: European League against Rheumatism Responder Index in 28 joints
HAQ-DI: Health Assessment Questionnaire–Disability Index
Ig: Immunoglobulin
LOCF: Last observation carried forward
MTX-IR: Methotrexate inadequate responder
NRI: Nonresponder imputation
OLE: Open-label extension
PY: Patient-year
Q4W: Every 4 weeks
RA: Rheumatoid arthritis
RCT: Randomized controlled trial
SAE: Serious adverse event
SD: Standard deviation
TEAb: Treatment-emergent antidrug antibody
TEAE: Treatment-emergent adverse event
TNF: Tumor necrosis factor
TNF-IR: Tumor necrosis factor antagonist inadequate responder
URI: Upper respiratory tract infection
UTI: Urinary tract infection
