# Supplementary material for: A 52-week, open-label study evaluating the safety and efficacy of tabalumab, an anti-B-cell–activating factor monoclonal antibody, for rheumatoid arthritis
Source: Arthritis Res Ther. 2014 Aug 29;16(4):415. doi: 10.1186/s13075-014-0415-2 (PMC4177512; doi:10.1186/s13075-014-0415-2)
Supplement: Additional file 1 — Ethical review boards that provided approval for this study. [file 13075_2014_415_MOESM1_ESM.docx]

**Additional file 1**

Ethical review boards that provided approval for this study are listed below:

- Quorum Review IRB
- Central Northern Adelaide Health Service Ethics of Human Research Committee (TQEH & LMEH)
- Ethics Committee of the Medical University Vienna
- Ethics Committee of Town Vienna
- Ethics Committee of the Medical University Vienna
- Komisja Bioetyczna przy Okregowej Izbie Lekarskiej
- National Ethics Committee for the Clinical Study of Medicines
- Comité d'Ethique Hospitalo - Facultaire Universitaire de Liège
- Egészségügyi Tudományos Tanács Klinikai Farmakológiai
- Institutional Ethics Committee - Nizams Institute of Medical Science
- Institutional Ethics Committee - Krishna Institute of Medical Sciences
- Hirabai Cowasji Jehangir Medical Research Institute and Jehangir Clinical Development Centre Ethics Committee
- Office of the Research Cell
- Comité de Ética em Pesquisa da UNICAMP
- Comité de Etica em Pesquisa com Seres Humanos da Pontificia Universidade Católica de Campinas (C.E.P.S.H.P.)
- Comitê de Ética da Faculdade de Medicina e Hospital Sâo Lucas – PUCRS
- Comitê de Ética em Pesquisa em Seres Humanos do Hospital de Clínicas da Universidade Federal do Paraná – CEP/HC/UFPR
- Comitê de Ética em Pesquisa Médica Humana e Animal
- Comitê de Ética em Pesquisa Humana e Animal do HGG
- University of Manitoba - Bannatyne Campus, Research Ethics Board
- Comité Etico Científico
- Comité de Ética e Investigación Servicio de Salud Valdivia
- Comité Etico Científico Servicio Salud Metropolitano Oriente
- CEC: Ethik-Kommission der Medizinischen Fakultat der Christian-Albrecht-Universitat zu Kiel, Universitats-Kinderklinik
- CEC: Ethik-Kommission der Medizinischen Fakultät der Christian-Albrechts-Uniersität zu Kiel
- Comite Bioetico para la Investigacion Clinica S.C.
- Comité de Ética e Investigación del Hospital Christus
- Comité de Ética e Investigación del Hospital Central
- Comité de Ética en Investigacion del Hospital Inovamed
- Comité de Ética del Instituto Jalisciense de Investigación
- Centro de Estudios de Investigacion Basica y Clinica SC
- Comité de Ética e Investigacion del Hospital y Clinica OCA
